# Effects of climate change on soil health resulting in an increased global spread of neglected tropical diseases

**DOI:** 10.1371/journal.pntd.0011378

**Published:** 2023-06-15

**Authors:** John Paul M. Wasan, Kishor M. Wasan

**Affiliations:** 1 Department of Plant Sciences, College of Agriculture and Bioresources, University of Saskatchewan, Saskatoon, Saskatchewan, Canada; 2 Department of Urologic Sciences, Faculty of Medicine & The Neglected Global Diseases Initiative, University of British Columbia, Vancouver, British Columbia, Canada; Emory University Department of Medicine, UNITED STATES

## Abstract

Although it is commonly accepted that climate change will increase the range and abundance of neglected tropical diseases (NTDs) through increased rainfall and temperature, the role of soil and influence of soil health on this effect is not well understood. We propose that understanding the influence of climate change on the physical, chemical, and biological characteristics of soils can explain how favourable environmental conditions for NTDs and vectors of NTDs to reproduce form. This, in turn, can assist local public health experts in predicting and managing the spread of NTDs. We also suggest that unlike unpredictable climatic factors, soil health can be directly managed through appropriate land use practices. This viewpoint seeks to start a discussion between soil scientists and healthcare professionals on how to achieve common goals and strategies required to manage the spread of NTDs.

## Introduction

Climate change has resulted in a significant increase in temperature, precipitation, humidity, and the frequency and intensity of storms [1–3]. These environmental changes have especially optimized the conditions for the emergence and spread of vector-borne neglected tropical diseases (NTDs) into novel regions of the world [1,2]. The increased emergence of NTDs due to this worldwide spread of such vectors should not be underestimated. The tiger mosquito (*Aedes albopictus*) can transmit many different types of arboviruses such as dengue fever, yellow fever, and West Nile virus, as well as nematode-borne parasitic diseases [4,5]. Phlebotomine sandflies are a vector of leishmaniasis. Under the complex effects of climate change, modelling the spread and annual cycling of these vectors has required additional hydrological and ecological data [6].

However, one underinvestigated aspect of these changes is the influence on landscapes and soils as vector habitats. It is possible that improved habitat conditions may enhance the emergence and spread of these vector-borne NTDs. If so, investigating relevant soil characteristics may improve models, identify novel areas of concern, and suggest land management practices to mitigate the spread of NTDs. It may be possible that soils that hinder the spread of disease are “healthy” soils, and soil health, like public health, can be improved and managed. Likewise, improved characterisation of regions at risk can better inform public health policy. To illustrate for the NTD community the role of soil in the spread of disease, we examine the concept of soil health and then consider the implications for example NTDs and vectors.

## Soil health

**Fig 1:**
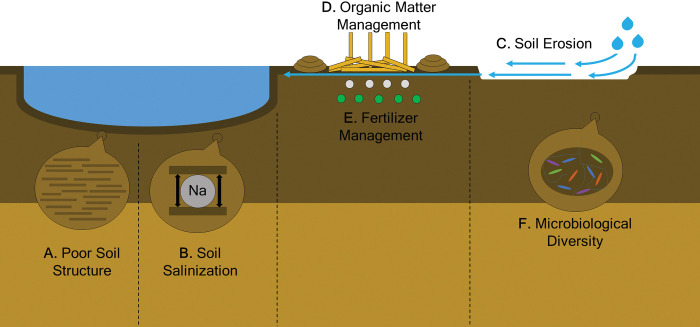
Soil health factors affecting the spread of NTDs. Standing water for breeding is a result of poor soil structure (**A**) and salinization (**B**) and can be enriched with organic matter and nutrients through soil erosion (**C**) interacting with poor organic matter (**D**) and fertilizer (**E**) management. Soil-dwelling vectors are affected by organic matter management (**D**) and the microbiome composition of the soil (**F**).

Soil health is generally defined in terms of ecosystem services (benefits of nature to humans), such as water retention, nutrient cycling, and supporting plant life [7]. Physically healthy soils are well aerated and have balanced moisture content. Fertile soils are high in organic matter and are abundant in plant nutrients. Chemically healthy soils are of moderate pH and not contaminated or saline. Finally, microbiologically healthy soils consist of diverse communities that efficiently cycle nutrients, form beneficial symbiotic relationships with plants, and suppress pathogens. Yet does climate change affect soil health in a manner that promotes the spread of NTDs? Is it possible to consider resistance to disease as a soil ecosystem service? Do the key soil characteristics vary by vector or disease? Identifying the soil health factors affecting the spread of NTDs (**Fig 1**) may be the key to their mitigation and prevention.

## Mosquito-borne diseases

There are numerous mosquito-borne diseases where newly formed ideal habitat conditions (i.e., increased precipitation, standing water, and eutrophication) have resulted in the emergence and spread of NTDs [6]. For this reason, dengue fever cases have been reported to increase in Taiwan, Australia, Mexico, Thailand, Peru, and other nations [6]. There are 3 key soil characteristics related to the formation of such habitats.

### Physical characteristics

The structure of a soil, the assembly of soil aggregates, greatly affects water movement and drainage. Fine-textured soils (i.e., clay), with very small pores, have high water retention and slow water drainage. This is especially true for clay soils with limited aggregation. More frequent extreme precipitation events expected with climate change may lead to increases in standing water [1], especially on these kinds of soils. Thus, when considering precipitation as a factor in modelling the spread of vectors, it is important to consider the local soil hydrology and texture. Further, improving soil structure through active land management (promoting root development to increase pore size) can aid in reducing standing water. Poor management, such as frequent soil compaction by machinery, can also degrade structure and impede water movement.

### Chemical characteristics

High concentrations of soluble salts, particularly sodium (sodic soils), can also affect soil structure and water drainage. Sodium acts as a dispersing agent in soils by widening the interior layers of clays, causing disaggregation. This reduces pore size and water flow. Soil sodicity depends on the rate of upward sodium-contaminated water movement (groundwater, evapotranspiration) versus the downward flux due to precipitation and irrigation. Therefore, with prolonged drought followed by sudden intense precipitation, coastal or inland standing water bodies can form on saline or sodic soils [8]. Rising sea levels have also been noted to increase the abundance of coastal saline water bodies, which increases selection pressure for and expands the range of saline-tolerant mosquito vectors [9]. In turn, this potentially could increase the prevalence of mosquito-borne diseases. In regions where soil salinity is extreme, models and experts should consider how precipitation, groundwater, and soil characteristics may affect the availability of standing water. Management practices such as using deep-rooted, high-water use plants, clean irrigation water, and deep tillage are effective remediation strategies to remove salts.

### Soil erosion

Increased intensity and frequency of precipitation events is predicted to lead to greater soil erosion [10]. Intensity is particularly important, as faster-moving raindrops and water can move larger aggregates and particles. While fine-textured soils are easily eroded, precipitation changes may also threaten medium-textured soils. Erosion has a significant effect on water quality, as it transports organic matter, fertilizers, and soil-adsorbed nutrients into water bodies, facilitating the breeding of vectors such as mosquitoes [11,12]. Therefore, manure and fertilizer placement method, application rate, and timing must consider the risk of runoff to water bodies. Nutrients, which are typically immobile, such as phosphorus, can have strong water quality impacts if mobilized. Controls both on-site and at water bodies, such as buffer strips (deep-rooted plants placed at field edges to filter off-site flow) and riparian areas (wetland vegetation with a similar effect), can reduce the impact of soil erosion on water quality and possibly on the spread of disease vectors.

## Sandfly-borne diseases

Phlebotomine sandflies reproduce in caves, human-made structures, and soil cervices, and larvae develop in humid, organic matter–rich environments [13]. This vector is particularly sensitive to human land use changes [1]. The increased emergence and spread of sandfly-borne diseases like leishmaniasis has also been linked to increases in temperature (increased metabolic activity accelerating the life cycle) and rainfall (greater availability of breeding sites) [1]. Soil and near-soil dwelling vectors are dependent on 2 major soil characteristics.

### Soil fertility

Many disease vectors, including sandflies, feed on organic matter during the larval stages [13]. The accumulation rate of organic matter (OM) depends on the input rate and recalcitrance versus the soil degradation rate. Ideal OM inputs for larvae include livestock manure, wildlife droppings, and plant residues, which are high quality (high nutrients relative to carbon) and not incorporated into the soil (slower decomposition) [13]. Yet in agriculture, soil incorporation is preferable to minimize losses and accelerate decomposition, especially in low-OM weathered tropical soils. Thus, management of OM to reduce availability to insects can benefit both soil and public health. While manure application on nutrient-poor soils is common, regions at risk of novel vectors should revaluate manure storage and management.

### Soil microbiology

As disease vectors enter new ecosystems, their interaction with their own soil-borne pathogens may also be altered by climate change. For example, sandflies are infected by the soil and water-dwelling pathogenic bacteria *Serratia marcescens*, which also targets *Leishmania* [14]. Where microbial diversity is stimulated, this potentially could competitively exclude pathogens, allowing expanding vectors to escape predation or reducing the abundance of sandfly-borne pathogens (dilution hypothesis). Conversely, greater overall diversity may directly lead to greater pathogen diversity (amplification hypothesis), resulting in exposure to new enemies or becoming a host to new pathogens. Where climate change reduces microbial diversity because of stress, vectors may again be released from competition or succumb to the same stress. Including interactions with vector competitors and pathogens in modelling may better project a vector’s ability to establish in a new environment.

## Soil-transmitted helminths

Land management for soil-based NTDs may be more challenging than soil-based vectors, because favourable conditions for crops and natural areas (high OM, moist soils, and available nutrients) may also benefit soil pathogens and other NTDs. For example, it is predicted that with increased soil humidity due to climate change, soil-transmitted helminth (STH) survival and spread will be enhanced [15]. As moist soil environments are especially important at the egg and larval stages to prevent desiccation [15], soils with high water retention capacity (fine-textured, high OM) are more likely to support STHs. However, especially in drought-prone regions, crop performance is often greatest on such soils. Avoiding use of contaminated irrigation water or manure, as well as modifying irrigation methods to limited prolonged saturated conditions, may be strategies to reduce soil-based NTD risk.

## Next steps

By considering physical, chemical, fertility, and microbiological characteristics of soil health, we may be able to better identify areas of concern related to the development and spread of NTDs. In addition to being an underrecognized reservoir for the production and spread of NTDs, unlike climatic factors, soil health can be actively managed to promote ecosystem services, which mitigate the spread of NTDs. By identifying key regions of concern where soil services are degraded, we can link land management to public health policy. Soil scientists, land managers, agronomists, farmers, and others are in a unique position to assist healthcare professionals in the fight against NTDs. Proactive, region-wide steps to simultaneously improve soil and human health are vital in light of climate change. The result can be increased agricultural yields and economic benefits, conservation of natural areas and resources, locally adapted drugs and therapies, and resistance to the spread of NTDs.
